# Children’s Health in Latin America: The Influence of Environmental Exposures

**DOI:** 10.1289/ehp.1408292

**Published:** 2014-12-05

**Authors:** Amalia Laborde, Fernando Tomasina, Fabrizio Bianchi, Marie-Noel Bruné, Irena Buka, Pietro Comba, Lilian Corra, Liliana Cori, Christin Maria Duffert, Raul Harari, Ivano Iavarone, Melissa A. McDiarmid, Kimberly A. Gray, Peter D. Sly, Agnes Soares, William A. Suk, Philip J. Landrigan

**Affiliations:** 1 Faculty of Medicine, University of the Republic of Uruguay, Montevideo, Uruguay; 2 Unit of Environmental Epidemiology, Institute of Clinical Physiology, National Research Council, Pisa, Italy; 3 Department of Public Health, Environmental and Social Determinants of Health Department, World Health Organization, Geneva, Switzerland; 4 Children’s Environmental Health Centre, Department of Paediatrics, University of Alberta, Edmonton, Alberta, Canada; 5 Unit of Environmental Epidemiology, Department of Environment and Primary Prevention, Istituto Superiore di Sanità, Rome, Italy; 6 Public Health Program, University of Buenos Aires, Buenos Aires, Argentina; 7 Blacksmith Institute, Buenos Aires, Argentina; 8 IFA Institute for Development of Production and Work Environment, Quito, Ecuador; 9 Division of Occupational Medicine, Department of Medicine, University of Maryland at Baltimore, Baltimore, Maryland, USA; 10 National Institute of Environmental Health Sciences, National Institutes of Health, Department of Health and Human Services, Research Triangle Park, North Carolina, USA; 11 Queensland Children’s Medical Research Institute, University of Queensland, Brisbane, Australia; 12 Pan American Health Organization, Washington, DC, USA; 13 Department of Preventive Medicine, and; 14 Arnhold Global Health Institute, Icahn School of Medicine at Mount Sinai, New York, New York, USA

## Abstract

**Background:**

Chronic diseases are increasing among children in Latin America.

**Objective and Methods:**

To examine environmental risk factors for chronic disease in Latin American children and to develop a strategic initiative for control of these exposures, the World Health Organization (WHO) including the Pan American Health Organization (PAHO), the Collegium Ramazzini, and Latin American scientists reviewed regional and relevant global data.

**Results:**

Industrial development and urbanization are proceeding rapidly in Latin America, and environmental pollution has become widespread. Environmental threats to children’s health include traditional hazards such as indoor air pollution and drinking-water contamination; the newer hazards of urban air pollution; toxic chemicals such as lead, asbestos, mercury, arsenic, and pesticides; hazardous and electronic waste; and climate change. The mix of traditional and modern hazards varies greatly across and within countries reflecting industrialization, urbanization, and socioeconomic forces.

**Conclusions:**

To control environmental threats to children’s health in Latin America, WHO, including PAHO, will focus on the most highly prevalent and serious hazards—indoor and outdoor air pollution, water pollution, and toxic chemicals. Strategies for controlling these hazards include developing tracking data on regional trends in children’s environmental health (CEH), building a network of Collaborating Centres, promoting biomedical research in CEH, building regional capacity, supporting development of evidence-based prevention policies, studying the economic costs of chronic diseases in children, and developing platforms for dialogue with relevant stakeholders.

**Citation:**

Laborde A, Tomasina F, Bianchi F, Bruné MN, Buka I, Comba P, Corra L, Cori L, Duffert CM, Harari R, Iavarone I, McDiarmid MA, Gray KA, Sly PD, Soares A, Suk WA, Landrigan PJ. 2015. Children’s health in Latin America: the influence of environmental exposures. Environ Health Perspect 123:201–209; http://dx.doi.org/10.1289/ehp.1408292

## Introduction

Physical, chemical, and biological hazards in the environment are responsible for > 24% of the global burden of disease and for 36% of all deaths in children worldwide (Prüss-Üstün and Corvalán 2006). The Pan American Health Organization (PAHO) estimates that nearly 100,000 children in the Americas < 5 years of age die each year from physical, chemical, and biological hazards in the environment (PAHO 2011). Hazardous environmental exposures in early life can produce disease in childhood and also influence health across the life span (Barker 2004).

The > 300 million children < 20 years of age who live in Latin America and the Caribbean (LAC) face a complex and rapidly changing array of environmental threats to health (PAHO 2011). These include the traditional hazards of indoor air pollution and contaminated drinking water (PAHO 2012a; WHO 2000). But additionally, children today are exposed to newer environmental threats such as urban air pollution, toxic synthetic chemicals, pesticides, heavy metals, unsustainable urban growth, hazardous wastes including electrical and electronic waste (e-waste), and global climate change [Barreto 2004; Barreto et al. 2012; PAHO 2012b; World Health Organization (WHO) and United Nations Environment Programme (UNEP) 2010].

The diseases caused by traditional environmental hazards are predominantly the ancient scourges of diarrhea and pneumonia plus a wide range of parasitic and other infectious diseases, especially malaria, dengue, and Chagas disease (Pronczuk et al. 2011). By contrast, modern environmental threats are linked mainly to chronic diseases: asthma, neurodevelopmental disorders, birth defects, obesity, diabetes, cardiovascular disease, mental health problems, and pediatric cancer (Haggerty and Rothman 1975; PAHO 2012b). This change in patterns of disease is termed the “epidemiological transition.” Countries passing through the epidemiological transition must deal simultaneously with epidemics of both infectious and chronic diseases.

The mix of environmental hazards and the diseases that they cause varies among countries in Latin America and even regionally within countries, depending on demographics, socioeconomic factors, urbanization, and degree of industrial development (Barreto 2004; De Maio 2011; PAHO 2007). Disparities in health status by race, ethnicity, and social class are powerful determinants of disease (PAHO 2011, 2012b). Some countries such as Argentina, Chile, and Uruguay have largely completed the epidemiological transition; some such as Brazil, Colombia, Costa Rica, Mexico, and Venezuela are passing through it now; and in others such as Ecuador and Guatemala the transition has been delayed, and infectious diseases are still the predominant causes of morbidity and mortality (Marinho et al. 2013).

Toxic chemicals are important causes of disease in Latin America. Many thousands of new chemicals and pesticides have been invented and spread into the environment over the past 50 years (Landrigan and Goldman 2011). Most did not previously exist in nature. Although not well publicized, this “chemical revolution” is one of the most important environmental changes that have transformed the developing world in the past half-century. Evidence of widespread exposure is provided by national surveys in industrially developed countries documenting measurable levels of > 100 synthetic chemicals in the bodies of virtually all persons (Centers for Disease Control and Prevention 2014). Children are exquisitely vulnerable to toxic chemicals [National Research Council (NRC) 1993; Prüss-Üstün and Corvalán 2006].

Urbanization is a powerful driving force in health and disease. Latin America is the most highly urbanized region of the world, with approximately 72% of the population living in urban centers [UN Habitat (United Nations Program for Human Settlement) 2012]. The fraction of the population residing in cities is rising steadily.

The climate of Latin America is also changing (Jenkins 1998; PAHO 2011). Deforestation is a major concern (PAHO 2012b). Climate change and deforestation can affect children’s health by leading to increased risk of floods, landslides, and extreme weather events and also by changing the distribution of insect vectors of disease (Patz et al. 1996).

To examine trends in pediatric disease and in the environment and to develop evidence-based strategies to improve child health and control environmental hazards, WHO developed a coordinated global program for research, education, and action in children’s environmental health (CEH) (Pronczuk et al. 2011; WHO and UNEP 2010). Formally launched in 2002 during the World Summit on Sustainable Development, the WHO CEH strategy aims to prevent disease and disability associated with chemical and physical threats to health. The WHO CEH program has evolved and developed through a series of conferences and consultations (see Supplemental Material, Appendix S1, “International and regional meetings on children’s environmental health convened by WHO”), and has achieved important successes (see Supplemental Material, Appendix S2, “Accomplishments of the WHO/PAHO program in children’s environmental health”). PAHO has led evolution of the CEH program in the Americas (PAHO 2011, 2012b).

In this review, we describe a plan for developing the WHO/PAHO strategic initiative in CEH in Latin America. Much of the material presented here was initially discussed in an International Scientific Conference: Environmental Health in the Political Agenda convened in Montevideo, Uruguay, on 22–24 March 2012. This conference honored the memory and life’s work of the late Uruguayan physician and global advocate for CEH, Jenny Pronczuk de Garbino. An editorial in this issue pays tribute to Dr. Pronczuk (Landrigan and Suk 2015).

## Specific Environmental Health Threats to Children in Latin America

*Indoor air pollution*. Indoor air pollution is the leading environmental threat to health in the Americas, being responsible for nearly 5% of healthy years of life lost and 7% of premature deaths (WHO 2014c). WHO estimates that in 2012, 7,500 deaths were attributable to indoor air pollution in children < 5 years of age in low- and middle-income countries of the Americas (WHO 2014c). In 2010, indoor air pollution ranked eighth among risk factors for chronic disease in Latin America (Lim et al. 2012).

Solid and biomass fuels are the major source of indoor air pollution, especially in rural areas. WHO estimates that in 2010, 10% of the population of Latin America relied on solid and biomass fuels for cooking and heating, largely in open fires or unvented stoves (WHO 2014b). The rural/urban ratio in use of solid fuels is 2.3 in countries in the lowest and, 11.7 in the highest quartile of the Human Development Index (http://hdr.undp.org/en/content/human-development-index-hdi). The Living Standards Measurement Survey (World Bank 2012) conducted in Guatemala demonstrated the highest reliance on solid fuel—up to 95% in some regions—among indigenous populations in rural areas.

Ten-fold disparities in death rates attributable to indoor air pollution are seen across Latin American countries and range from a high of 14 in the least developed countries, down to 0.3 for high-income countries (WHO 2014b). Thus in 2012, ≥ 50% of the populations of Guatemala, Haiti, Honduras, and Nicaragua used solid fuels as their main energy source (WHO 2014b). Even in urban areas of the less-developed countries of the Americas, a high percentage of the population relies on solid fuels (Soares da Silva et al. 2013; WHO 2014b).

Since 1990 the region has experienced a steady decline in the percentage of the population using solid fuels and the size of the population exposed (Bonjour et al. 2013). Some countries can make the transition to cleaner fuels, but others will likely continue to use solid fuels because of lack of infrastructure and the high costs associated with transition to gas and electricity.

Interventions are underway, especially in areas of the Americas, to introduce new cookstoves that use solid fuels more efficiently and are less polluting. A major study of this intervention in Guatemala found that using clean stoves was associated with a 30% reduction in severe pneumonia among children < 18 months of age (Smith et al. 2011). A study in Peru found that using the new cookstoves significantly reduced sleep and respiratory symptoms in children 2–14 years of age, but only in households that used the less-polluting stoves exclusively and with adequate maintenance (Accinelli et al. 2014).

*Ambient air pollution*. Children in the mega-cities of Latin America—Mexico City, Mexico; Santiago, Chile; and São Paulo, Brazil—are exposed to high levels of air pollution (WHO 2014d). This pollution is associated with poor respiratory health (de Medeiros et al. 2009; Romieu et al. 2003). Even in smaller cities, ambient air pollution from stationary and mobile sources has also been shown to negatively affect children’s respiratory health (Wichmann et al. 2009).

Important research on air pollution and its health effects has been undertaken in Latin America (PAHO 2012b). de Medeiros et al. (2009) conducted a case–control study in 14 districts of São Paulo that showed that infants born to women in regions with the highest levels of traffic-related pollution (upper quintile) had an almost 50% increased risk of early neonatal death compared with those living in areas with the lowest pollution [adjusted odds ratio (OR) = 1.47; 95% confidence interval (CI): 0.67, 3.19]. Exposure to traffic-related pollution in Latin America has been associated with increased otitis media (Brauer et al. 2006), increased asthma, more severe asthma symptoms, and lower lung function (Barraza-Villarreal et al. 2011; Romieu et al. 2008; Wichmann et al. 2009).

The source of air pollution has been shown to influence the resulting health effects. In a study from La Plata, Argentina, children exposed to industrial pollution near a major petrochemical refining area had a pattern of respiratory impairment that was different from that of children living in regions of the city with predominantly traffic-related pollution (Wichmann et al. 2009). Risk of asthma (OR = 2.76; 95% CI: 1.96, 3.89), acute asthma exacerbations (OR = 1.88; 95% CI: 1.25, 1.83), and wheeze (OR = 1.93; 95% CI: 1.39, 2.67) were all increased among children living in the industrial area. By contrast, low lung function and airway obstruction were more common among children with exposure to air pollutants of automotive origin.

*Lead*. The Americas account for one-third of global lead production (PAHO 2011), and lead is widespread in the Latin American environment. Lead is responsible for 1% of the global burden of disease (Fewtrell et al. 2004). About 99% of all children affected by exposure to lead live in low- and middle-income countries (Suk et al. 2003). Children living in poverty are most likely to be exposed to lead (Rojas et al. 2003; Seijas and Squillante 2008).

Smelters and battery factories are important sources of lead exposure in Latin America, especially for children living close to these plants. A mean blood lead level (BLL) of 17.1 μg/dL, with a range from 2.0–36.2 μg/dL, was reported in a population living near an industrial facility in Uruguay (Mañay et al. 2008). Other sources of exposure include e-waste, lead paint, battery recycling, lead pipes, and the burning of solid waste (Anticona et al. 2011; Coria et al. 2009; Manzanares-Acuña et al. 2006; Sánchez-Cortez et al. 2003). Contaminated waste sites and dumps containing industrial, metallurgic, and mining wastes such as La Oroya and el Callao in Peru, Abra Pampa in Argentina, and La Teja in Uruguay are responsible for some of the most severe lead exposure scenarios in the region (Espinoza et al. 2003; Garcia and Mercer 2003; Rubio-Andrade et al. 2011; Schütz et al.1997).

Public health interventions to reduce lead exposure have begun to yield positive results in Latin America. Although there is no uniform epidemiologic database that tracks BLLs across the region, protocols to reduce lead exposure and to monitor children’s lead exposure are being put in place in various countries such as Mexico (Recio-Vega et al. 2012), Peru (Ministerio de Salud Publica, Republica del Peru 2007), Argentina (Ministerio de Salud, Republica de Argentina 2013), and Uruguay (Ministerio de Salud Publica, Republica Oriental del Uruguay 2011). CEH units (WHO 2010b) and referral centers for diagnosis and prevention of lead poisoning have been established (Laborde 2004). Some countries such as Chile (Ministerio de Salud, Comision Nacional del Medio Ambiente, Gobierno de Chile 2009) and Uruguay [Ministerio de Vivienda, Ordinamiente Teritorial y Medio Ambiente, Republica Oriental del Uruguay (MVOTMA) 2012] have established legislation regulating battery recycling. Argentina (Ministerio de Salud, Republica de Argentina 2009), Brazil, Chile (Biblioteca del Congreso Nacional de Chile, Ministerio de Salud, Gobierno de Chile 1997), and Uruguay (MVOTMA 2011) have established national regulations limiting the lead content of lead in residential paints.

The phase-out of lead from gasoline has been a particularly important advance (Franco-Netto et al. 2003). Until leaded gasoline was banned, BLLs > 10 μg/dL were found in 10–40% of children in urban areas in Latin America, with the highest levels in countries that used leaded gasoline. Children’s BLLs in Latin America have declined following the removal of lead from gasoline (Romieu et al. 1995; Sánchez-Cortez et al. 2003).

Further research is needed in Latin America to identify additional sources of environmental exposure to lead, to screen children at risk, and to guide educational, nutritional, environmental, and medical interventions that will further reduce children’s exposures to lead (Kordas et al. 2010; Vega-Dienstmaier et al. 2006).

*Mercury*. Mercury contamination found in many areas of Latin America poses serious threats to children’s health. Artisanal gold mining uses mercury to remove gold from ore and is a major source of this contamination (Gibb and O’Leary, 2014; Wade 2013). Occupational and para-occupational exposures to metallic mercury, including exposures to children, are extensive in artisanal gold mining, and neither preventive nor protective measures are regularly used (Bose-O’Reilly et al. 2008; Cordy et al. 2011). Formation of highly neurotoxic methyl mercury from metallic mercury that enters streams and rivers is a further hazardous consequence of artisanal gold mining (Grandjean et al. 1999).

A study of pediatric mercury exposure in the mining area of Camilo Ponce Enriquez, Ecuador, within the framework of PHIME (Public Health Impact of long-term, low-level Mixed Element exposure in susceptible population strata) reported a geometric mean mercury concentration in blood of 3.23 μg/L (range, 1.0–25 μg/L) among 69 children 7–10 years of age (Hrubá et al. 2012; Pawlas et al. 2013). Additional studies in urban and rural communities around this mining area also demonstrated elevated levels of mercury in blood (Hrubá et al. 2012; Pawlas et al. 2013).

*Asbestos*. Global consumption of asbestos has declined over the past 25 years as a steadily increasing number of countries have recognized the health hazards and taken legal action to restrict or ban asbestos use. The use of asbestos has been virtually eliminated in most industrially developed countries. Current annual global consumption is about 2 million tons, and virtually all of that use takes place in developing countries (Virta 2009).

In Latin America, progress is being made in reducing asbestos use. Argentina, Chile, Uruguay, and Honduras have all banned asbestos. Consumption is declining in other countries, even where it is not actually illegal. But progress is slow and not necessarily irreversible. Indeed some sources report rising asbestos consumption in a handful of countries such as Mexico and El Salvador (Roselli 2014).

The two largest users of asbestos in Latin America are Brazil and Colombia, and both are also asbestos producers (Castleman 1999). In 2012, Brazil produced 306,500 tons of asbestos and was the world’s third largest producer (Virta 2012). Annual production in Colombia in 2003 and 2004 was an estimated 60,000 tons (Roselli 2014). About 80% of the asbestos used in Brazil is consumed in the form of asbestos cement: for roof tiles and roofing panels, wall-board, and domestic and industrial water tanks (Berman 1986).

*Arsenic*. Arsenic is a metalloid element that occurs naturally in the earth’s crust in organic and inorganic forms (Naujokas et al. 2013). High concentrations are also released into the environment by polluting industries. Worldwide, drinking water is the principal source of arsenic exposure, but inhalation can be an important exposure route for people living near polluting industries as well as for children working in mining and smelting. Arsenic is widely distributed in the environment of Latin America, and elevated levels have been documented in drinking water in areas of several countries, most notably Chile, Argentina, and Mexico (Bundschuh et al. 2012).

Inorganic arsenic is classified by the International Agency for Research on Cancer (IARC) as a class I human carcinogen (IARC 2012). Inorganic arsenic compounds have been shown in human studies to cause cancer of the lung, urinary bladder, and skin. Also, positive associations have been observed between inorganic arsenic compounds and cancer of the kidney, liver, and prostate. Inorganic arsenic is also associated with cardiovascular disease.

Prenatal and pediatric exposures to arsenic in Latin America have been linked to adverse perinatal, childhood, and adult health outcomes. Some of the most detailed studies of these effects were conducted in Antofagasta in northern Chile, where the arsenic concentration in the public drinking-water supply rose sharply between 1958 and 1970 because of a contaminated water source.

Perinatal arsenic exposures in Antofagasta were linked to increases in late fetal, neonatal, and postneonatal mortality (Hopenhayn-Rich et al. 2000). In childhood, an increased number of skin lesions were reported in exposed children, and chronic cough was reported by 38.8% of children with arsenic-related skin lesions, compared with 3.1% of children with normal skin (Borgoño et al. 1977). Last, a cancer epidemiology study in Antofagasta found that arsenic exposures in early life were strongly associated with increased mortality from bladder cancer, laryngeal cancer, liver cancer, and chronic renal disease in adults < 50 years old (Smith et al. 2012). A similar pattern of cancer risk was demonstrated in people chronically exposed to elevated levels of arsenic in groundwater in northern Argentina (Aballay et al. 2012).

Several well-conducted prospective epidemiological studies have linked pediatric and prenatal exposures to arsenic with decreases in learning ability and neurodevelopmental abnormalities (von Ehrenstein et al. 2007; Wasserman et al. 2004). More recently, a study of children living in an area of Torreon, Mexico, contaminated with both arsenic and lead, showed that arsenic contamination can affect children’s cognitive development, independent of any effect of lead (Rosado et al. 2007).

Control of arsenic exposure can reduce health risks, but studies in Antofagasta demonstrate that the elevated rates of cancer can persist for many years after cessation of high-dose exposure (Steinmaus et al. 2013).

*Pediatric cancer*. Children around the world, including children in Latin America are at risk of exposure to multiple carcinogens in air, water, food, and soil. These exposures can cause pediatric cancer, which accounts for about 1% of all reported cancers (Buka et al. 2007) and also lead to cancers in adult life (Smith et al. 2012). Children are highly vulnerable to environmental carcinogens because of their unique patterns of exposure, rapid growth and development, dynamic physiology, and long life expectancy (NRC 1993).

A population-based case–control epidemiologic study in Costa Rica identified an association between parental exposures to pesticides during and before pregnancy and increased risk of childhood leukemia (Monge et al. 2007). A small case–control study in Mexico also found some evidence for an association between childhood acute leukemia and domestic or garden use of pesticides in the 3 months before pregnancy (Hernández-Morales et al. 2009). Last, a large hospital-based case–control study showed associations between using pesticides during pregnancy and acute lymphoid leukemia and acute myeloid leukemia (Ferreira et al. 2013).

Monitoring releases from industry to air, water, and land and making this information public is an important strategy for controlling environmental exposures to carcinogens and other toxic chemicals (UN 2013). Long used in North America and Europe, this approach is now spreading to Latin America. In 2006, the Registro de Emisiones y Transferencia de Contaminantes in Mexico collected the first emissions data, which included 104 chemicals from > 1,000 industries, and made the data public (Commission for Environmental Cooperation 2013).

*Pesticides*. Pesticide use is widespread in Latin America. According to PAHO, the region spends nearly US$3 billion each year on pesticides (PAHO 2011). In Central America, PAHO has tracked a steady increase in acute pesticide poisoning cases each year for the past two decades, and this trend closely parallels upward trends in pesticide imports (Henao and Arbelaez 2002).

Acute pesticide poisoning is widespread in Latin America, and PAHO estimates that acute pesticide poisoning cases are underreported by 50–80% (PAHO 2011). Between 2003 and 2006, the Montevideo Poison Control Center (http://www.ciat.hc.edu.uy) received 18,110 consultations involving people < 15 years of age, and > 15% of these consultations were related to pesticides (Juanena et al. 2011). Epidemiologic studies in Ecuador, Costa Rica, and Nicaragua indicate that organophosphate and carbamate insecticides are the most frequent source of acute pesticide poisoning in people of all ages (Bovi de De Pascuale et al. 1995; Wesseling et al. 1997, 2005).

Acute pesticide poisoning in children can result from nonintentional ingestion of pesticides stored in medicine or soft drink bottles or when pesticide containers are reused for storing drinks or food (Juanena et al. 2011). Dermal exposure occurs when parents use the pesticide to treat scabies or head lice; lindane and the pyrethroids are the pesticides most commonly used in this application (Rodríguez et al. 2007). Studies of knowledge, attitudes, and practices indicate that unsafe use of pesticides is a common problem in Latin American countries (Cervantes Morant 2010).

Chronic exposure to pesticides is also widespread among children in Latin America. Pathways of chronic exposure include drift from sprayed fields, persistence in sprayed homes, and water or food contamination (de Carvalho Dores and De-Lamonica-Freire 2001; Dutra Caldas and de Souza Kenupp 2000).

Latin American children are occupationally exposed to pesticides because they represent a substantial part of the agricultural workforce and begin work at a young age (González-Andrade et al. 2010; Tomasina and Laborde 2010). Decreases in cholinesterase levels have been reported over the course of a growing season in children living in rural areas (Bovi de De Pascuale et al. 1995; Henao and Arbelaez 2002). Parental occupational exposures can indirectly influence child health when toxicants are carried home on parents’ contaminated work clothes or other objects. Many toxicants encountered by women at work can also be transmitted to their infants via breast milk (McDiarmid and Gehle 2006; Mutanen and Hemminki 2001).

The developmental toxicity of pesticides has been studied in Latin America. Alterations in fetal growth and length of gestation have been reported in children born to mothers with agricultural exposure to pesticides during pregnancy (Cecchi et al. 2012; Souza et al. 2005, 2006). Risk factors for these effects included living near treated fields, storage of pesticides at home, and direct or accidental contact with pesticides (Benítez-Leite et al. 2009). Investigations in Ecuador found that prenatal exposure to pesticides is associated with severe adverse effects on brain development in children, even at low levels of exposure (Harari et al. 2010). Handal et al. (2007) showed that residence in communities with high potential for exposure to organophosphate and carbamate pesticides was associated with poorer neurobehavioral development. Malnourished populations may be particularly vulnerable to these neurobehavioral effects (Handal et al. 2007). It has been reported that exposure to pesticide mixtures in agriculture-intensive areas in Uruguay may increase risk for early-onset respiratory disease (Laborde et al. 2006). Occupational contact with pesticides and other chemicals is another route of prenatal exposure. Physiologic changes that occur in pregnancy may increase the toxicity of certain chemical exposures (McDiarmid and Gehle 2006; Paul and Himmelstein 1988).

Biomarkers have been used to study pesticide exposure in Latin American children. A recent study in Argentina evaluated saliva sampling as a tool for biological monitoring and suggested that salivary carboxylesterase levels might be a sensitive, noninvasive biomarker for assessing pediatric exposure to organophosphate pesticides (Bulgaroni et al. 2012).

*Hazardous waste sites*. Toxic waste sites are a major, and heretofore underrecognized, global health problem, especially in developing countries (Naujokas et al. 2013). PAHO estimates that 500,000 people in the Americas live on or close by a hazardous waste site (PAHO 2011).

Recent studies have found that hazardous waste sites are major contributors to the burden of disease in low- and middle-income countries and are responsible for a disease burden comparable to those caused by outdoor air pollution and malaria (Chatham-Stephens et al. 2013). Exposures in early life to pesticides and toxic chemicals from hazardous waste sites may cause severe developmental defects and entail lifelong health consequences (NRC 1993).

*E-waste.* E-waste disposal sites represent a relatively new and rapidly growing type of hazardous waste site in Latin America and around the world (Araújo et al. 2012; Cyranek and Silva 2010; Gavilán-García et al. 2009; Steubing et al. 2010). More than 45 million tons (Noel-Brune et al. 2013) of e-waste were generated globally in 2012, and these numbers are expected to rise in coming years (UN 2012; Zoeteman et al. 2010). Significant amounts of e-waste are exported from developed countries to developing countries, including Latin American countries, where recycling of valuable compounds from e-waste, such as copper and gold, has evolved into an important business, predominantly in the informal sector (Gunsilius et al. 2011; UNEP 2010).

E-waste contains a vast number of hazardous chemicals including lead, cadmium, mercury, nickel, barium, and lithium, as well as persistent organic pollutants such as polychlorinated biphenyls (PCBs) and brominated flame retardants (Cyranek and Silva 2010; Lundgren 2012; UNEP 2010; WHO 2010c). Unsafe recycling activities such as open burning and acid leaching of e-waste produce toxic combustion products including dioxin-like PCBs, and polychlorinated dibenzo-dioxins and -furans (PCDD/Fs) (Frazzoli et al. 2010).

E-waste may pose significant health risks to children, especially child workers (Wu et al. 2010). Children may also be confronted with hazardous substances through take-home exposures and home-based e-recycling workshops. Children at e-waste sites have been reported to display elevated levels of multiple toxic chemicals including lead (Huo et al. 2007; Xu et al. 2012), nickel (Zheng et al. 2013), manganese (Zheng et al. 2013), and chromium (Li et al. 2008a). These exposures are associated with a range of adverse health effects, including injury to children’s immune, cardiovascular, gastrointestinal, renal, endocrine, and hematological systems; adverse birth outcomes, such as stillbirth and lower APGAR scores; reduced pulmonary function; effects on child physical growth; and altered neurobehavioral development (Li et al. 2008a, 2008b; Liu et al. 2011; WHO 2010a; Yang et al. 2013; Zheng et al. 2013).

Analyses of e-waste in Latin America project increasing regional levels of e-waste generation in future years (Cyranek and Silva 2010; Gavilán-García et al. 2009; Steubing et al. 2010) and discuss the need for development of comprehensive national systems for e-waste management (de Oliveira et al. 2012).

*Regional climate change and children’s health*. Despite the global nature of climate change, it produces highly variable local impacts and specific effects. Children are especially vulnerable to these effects (Sheffield and Landrigan 2011).

Ecuador provides a case study within Latin America of the varied local effects of climate change. Ecuador is one of the most highly biodiverse countries in the world, with its tropical forest (Amazonia), Andes, and coastal regions each having a distinct ecosystem (Bass et al. 2010). In the vulnerable ecosystem of Amazonia, highly aggressive wood production and petroleum extraction have led to deforestation (Ministerio del Ambiente, República del Ecuador 2003), which in concert with the increased rainfall associated with global climate change has produced increased risk of landslides and flooding. In the Andes, glaciers are melting so fast that future water supplies are threatened and desertification is accelerated (Bradley et al. 2006; Francou et al. 2011; PAHO 2011). On the coast, extreme heat events are increasing in frequency (Ministerio del Ambiente, República del Ecuador 2012).

Increased ultraviolet radiation from depletion of stratospheric ozone is a consequence of climate change observed to especially affect people in the high Andes of Ecuador (PAHO 2012b; Pinault and Hunter 2011). Skin cancer is now the second most frequent form of cancer in Ecuador (Cueva and Yépez 2009).

Increased evaporation of pesticides because of increased thermal stress is yet another consequence of climate change (Chen and McCarl 2001; Noyes et al. 2009; Sciedeck et al. 2007). This effect has been documented in Latin America at banana plantations, which are heavy consumers of multiple pesticides. To evaluate children’s exposure to chlorpyrifos in villages situated near banana plantations and plantain farms, a cross-sectional study in Costa Rica examined urinary levels of 3,5,6-trichloro-2-pyridinol (TCPy), a biomarker for chlorpyrifos exposure, in 140 mostly indigenous children. The study targeted two villages that were situated close to plantations that used chlorpyrifos and one comparison village with mainly organic production. The main finding was that children from the villages near the plantations that used pesticides had significantly higher urinary TCPy concentrations than did children from the control village. Chlorpyrifos was detected in 30% of the environmental samples as well as in 92% of the hand/foot wash samples in the villages near the pesticide-using plantations (van Wendel de Joode et al. 2012).

Adverse socioeconomic conditions such as high rates of poverty, inadequate industrial development, low productivity in traditional agriculture, and lack of safe drinking water can magnify vulnerability to climate change in Latin American countries (Harari and Harari 2006). The lack of public services that is common in some socially isolated areas further increases societal vulnerability (McMichael et al. 2004; PAHO 2011; PAHO and WHO 2007).

## Conclusions

Chronic diseases are on the rise among children in Latin America. These diseases include asthma, neurodevelopmental disorders, birth defects, obesity, diabetes, cardiovascular disease, mental health problems, and pediatric cancer. Their increase is part of a profound regional shift in patterns of morbidity and mortality—an epidemiological transition (Barreto 2004; Barreto et al. 2012; Marinho et al. 2013). Although the pace of this transition is uneven and varies from country to country depending on degree of industrial development, urbanization, and socioeconomic status, the trend away from the ancient infectious diseases and toward chronic, noncommunicable disease is clear and is accelerating in Latin America and around the world (UN 2011).

Concomitant with the epidemiological transition, far-reaching environmental changes have swept Latin America. These include urbanization, industrialization, changes in patterns of land use, deforestation, and the effects of regional climate change. Environmental degradation has occurred in many areas.

Toxic chemicals in the environment have become an important problem and are recognized as major causes of disease and disability among children in Latin America. Chemicals including lead, mercury, industrial chemicals, air pollution, pesticides, and chemicals from e-waste have been documented to be responsible for a wide range of illnesses among children in the region (Hopenhayn-Rich et al. 2000; Hrubá et al. 2012; Mañay et al. 2008; Romieu et al. 2003; Wesseling et al. 2005; WHO 2014c).

To confront the crisis of chronic disease of environmental origin among children in Latin America, WHO, including PAHO, plans to build on current efforts and concentrate resources on a carefully selected short list of the most pressing problems, which include the following:

Indoor and outdoor air pollution, which together led to > 7 million deaths worldwide in 2012—131,000 of them in low- and middle-income countries in the Americas (WHO 2014a).
Water pollution (PAHO 2012a; WHO 2000).
Toxic chemical hazards (WHO 2010d, 2014a).

WHO, including PAHO, plans to move forward in CEH in the following areas:

Develop tracking data for Latin America on trends on CEH, including updated estimates of the burden of disease due to environmental factors.
Develop a network of Collaborating Centres, collaborators, and experts from research institutions, academia, health and environment sectors, policy makers, governments, and nongovernmental organizations in the LAC countries and worldwide to ensure that the best experts and key players are involved (Sly et al. 2014).
Conduct studies of the economic costs of asthma, obesity, and neurodevelopmental problems as well as studies of the economic benefits that will accrue to societies worldwide through control of these hazards.
Support the development of policies prioritized on the global burden of disease and economic costs of exposures of environmental exposures.
Promote biomedical research in CEH including toxicological and biomarker research, multi-year longitudinal birth cohort studies, and shorter-term epidemiological studies of pressing problems such as endocrine disruptors, environmental causes of neurodevelopmental disorders, and environmental causes of asthma. This research will provide a blueprint for evidence-based prevention and environmental intervention.
Build regional capacity in CEH, focusing on the health sector, addressing new audiences, and promoting tools such as environmental health questionnaires (the “Green Page”; WHO 2012) and referral centers such as CEH units (WHO 2010b).
Develop a platform for dialogue with other UN agencies, such as United Nations Environment Programme and UNICEF (United Nations Children’s Fund), to share information and work together in common objectives.

In the 21st century, the protection of children’s health and the prevention of pediatric disease of environmental origin in Latin America and around the world will require a highly focused, visible, and sustainably funded strategy that focuses on the most important health threats.

## Supplemental Material

(207 KB) PDF

## References

[r1] Aballay LR, del Pilar Díaz M, Francisca FM, Muñoz SE (2012). Cancer incidence and pattern of arsenic concentration in drinking water wells in Córdoba, Argentina.. Int J Environ Health Res.

[r2] Accinelli RA Llanos O López LM Pino MI Bravo YA Salinas V 2014 Adherence to reduced-polluting biomass fuel stoves improves respiratory and sleep symptoms in children. BMC Pediatr 14 12; 10.1186/1471-2431-14-12 24433576 PMC3898254

[r3] Anticona C, Bergdahl IA, Lundh T, Alegre Y, Sebastian MS (2011). Lead Exposure in indigenous communities of the Amazon basin, Peru.. Int J Hyg Environ Health.

[r4] Araújo MG, Magrini A, Mahler CF, Bilitewski B (2012). A model for estimation of potential generation of waste electrical and electronic equipment in Brazil.. Waste Manag.

[r5] Barker DJ (2004). The developmental origins of adult disease.. J Am Coll Nutr.

[r6] Barraza-Villarreal A, Escamilla-Nuñez MC, Hernández-Cadena L, Texcalac-Sangrador JL, Sienra-Monge JJ, Del Río-Navarro BE (2011). Elemental carbon exposure and lung function in schoolchildren from Mexico City.. Eur Respir J.

[r7] Barreto ML (2004). The globalization of epidemiology: critical thoughts from Latin America.. Int J Epidemiol.

[r8] Barreto SM, Miranda JJ, Figueroa JP, Schmidt MI, Munoz S, Kuri-Morales (2012). Epidemiology in Latin America and the Caribbean: current situation and challenges.. Int J Epidemiol.

[r9] Bass MS Finer M Jenkins CN Kreft H Cisneros-Heredia DF McCracken SF 2010 Global conservation significance of Ecuador’s Yasuni National Park. PLoS One 5 1 e8767; 10.1371/journal.pone.0008767 20098736 PMC2808245

[r10] Benítez-Leite S, Macchi ML, Acosta M (2009). Congenital malformations associated with toxic agricultural chemicals.. Arch Pediatr Urug.

[r11] Berman D (1986). Asbestos and health in the third world: the case of Brazil.. Int J Health Serv.

[r12] Biblioteca del Congreso Nacional de Chile, Ministerio de Salud, Gobierno de Chile. (1997). Fija limite máximo permisible de plomo en pinturas que indica. dto. nº 374/97 [in Spanish].. http://www.sernac.cl/wp-content/uploads/2012/12/DTO-374_Fija-limite-maximo-permisible-de-plomo-en-pinturas-que-indica-25-AGO-1997.pdf.

[r13] Bonjour S Adair-Rohani H Wolf J Bruce NG Mehta S Prüss-Ustün A 2013 Solid fuel use for household cooking: country and regional estimates for 1980–2010. Environ Health Perspect 121 784 790; 10.1289/ehp.1205987 23674502 PMC3701999

[r14] Borgoño JM, Vicent P, Venturino H, Infante A (1977). Arsenic in the drinking water of the city of Antofagasta: epidemiological and clinical study before and after the installation of a treatment plant.. Environ Health Perspect.

[r15] Bose-O’Reilly S, Lettmeier B, Gothe RM, Beinhoff C, Siebert U, Drasch G (2008). Mercury as a serious health hazard for children in gold mining areas.. Environ Res.

[r16] Bovi de De Pascuale G, Ruggeri MA, Singh J. (1995). Uso de plaguicidas en la provincia de Jujuy [in Spanish].. Proyección, Publicación especializada del colegio de Ingenieros de Jujuy.

[r17] Bradley RS, Vuille M, Diaz HF, Vergara W (2006). Climate change. Threats to water supplies in the tropical Andes.. Science.

[r18] Brauer M Gehring U Brunekreef B de Jongste J Gerritsen J Rovers M 2006 Traffic-related air pollution and otitis media. Environ Health Perspect 114 1414 1418; 10.1289/ehp.9089 16966098 PMC1570088

[r19] Buka I, Koranteng S, Osornio-Vargas AR (2007). Trends in childhood cancer incidence: review of environmental linkages.. Pediatr Clin North Am.

[r20] Bulgaroni V, Rovedatti MG, Sabino G, Magnarelli G (2012). Organophosphate pesticide environmental exposure: analysis of salivary cholinesterase and carboxilesterase activities in preschool children and their mothers.. Environ Monit Assess.

[r21] Bundschuh J, Litter MI, Parvez F, Román-Ross G, Nicolli HB, Jean JS (2012). One century of arsenic exposure in Latin America: a review of history and occurrence from 14 countries.. Sci Total Environ.

[r22] Castleman B (1999). Global corporate policies and international “double standards” in occupational and environmental health.. Int J Occup Environ Health.

[r23] Cecchi A, Rovedatti MG, Sabino G, Magnarelli GG (2012). Environmental exposure to organophosphate pesticides: assessment of endocrine disruption and hepatotoxicity in pregnant women.. Ecotoxicol Environ Saf.

[r24] Centers for Disease Control and Prevention. (2014). Fourth National Report on Human Exposure to Environmental Chemicals. Updated Tables, August 2014.. http://www.cdc.gov/exposurereport/.

[r25] Cervantes Morant R. (2010). Plaguicidas en Bolivia: sus implicaciones en la salud, agricultura y medio ambiente [in Spanish]. REDESMA 4.. http://www.cebem.org/cmsfiles/articulos/REDESMA_09_art02.pdf.

[r26] Chatham-Stephens K Caravanos J Ericson B Sunga-Amparo J Susilorini B Sharma P 2013 Burden of disease from toxic waste sites in India, Indonesia, and the Philippines in 2010. Environ Health Perspect 121 791 796; 10.1289/ehp.1206127 23649493 PMC3702002

[r27] Chen CC, McCarl BA (2001). An investigation of the relationship between pesticide usage and climate change.. Clim Change.

[r28] Commission for Environmental Cooperation. (2013). Tracking Pollutant Releases and Transfers in North America.. http://www.cec.org/Page.asp?PageID=924&SiteNodeID=1016&AA_SiteLanguageID=1.

[r29] Cordy P, Veiga MM, Salih I, Al-Saadi S, Console S, Garcia O (2011). Mercury contamination from artisanal gold mining in Antioquia, Colombia: the world’s highest per capita mercury pollution.. Sci Total Environ.

[r30] Coria C, Cabello A, Tassara E, López E, Rosales H, Pérez M (2009). Efectos clínicos a largo plazo en niños intoxicados con plomo en una región del sur de Chile [in Spanish].. Rev Med Chile.

[r31] Cueva P, Yépez, J, eds. (2009). Registro Nacional de Tumores. Epidemiologia del Cáncer en Quito 2003-2005. XIV Edición [in Spanish].

[r32] Cyranek G, Silva U, eds. (2010). Los residuos electrónicos: un desafío para la sociedad del conocimiento en América Latina y el Caribe [in Spanish].

[r33] de Carvalho Dores EFG, De-Lamonica-Freire EM (2001). Aquatic environment contamination by pesticides. Case study: water used for human consumption in Primavera do Leste, Mato Grosso—preliminary analyses [in Portuguese].. Quim Nova.

[r34] De Maio FG 2011 Understanding chronic non-communicable diseases in Latin America: towards an equity based research agenda. Global Health 7 36; 10.1186/1744-8603-7-36 21981767 PMC3193810

[r35] de Medeiros AP Gouveia N Machado RP de Souza MR Alencar GP Novaes HM 2009 Traffic-related air pollution and perinatal mortality: a case–control study. Environ Health Perspect 117 127 132; 10.1289/ehp.11679 19165399 PMC2627856

[r36] de Oliveira CR, Bernardes AM, Gerbase AE (2012). Collection and recycling of electronic scrap: a worldwide overview and comparison with the Brazilian situation.. Waste Manag.

[r37] Dutra Caldas E, de Souza Kenupp LC (2000). Assessment of the chronic risk for ingestion of pesticide residues in the Brazilian diet [in Portuguese].. Rev Saude Publica.

[r38] Espinoza R, Hernández-Avila M, Narciso J, Castañaga C, Moscoso S, Ortiz G (2003). Determinants of blood-lead levels in children in Callao and Lima metropolitan area.. Salud Publica Mex.

[r39] Ferreira JD Couto AC Pombo-de-Oliveira MS Koifman S Brazilian Collaborative Study Group of Infant Acute Leukemia. 2013 *In utero* pesticide exposure and leukemia in Brazilian children < 2 years of age. Environ Health Perspect 121 269 275; 10.1289/ehp.1103942 23092909 PMC3569673

[r40] Fewtrell LJ, Prüss-Ustün A, Landrigan P, Ayuso-Mateos JL (2004). Estimating the global burden of disease of mild mental retardation and cardiovascular diseases from environmental lead exposure.. Environ Res.

[r41] Franco-Netto G, Alonzo HG, Cancio J, Jost M, de Souza-Oliveira S (2003). Human health risk reduction due to lead exposure in Brazil.. Salud Publica Mex.

[r42] Francou B, Cáceres B, Villacís M, Basantes R, Maisincho L, Galárraga R, et al. (2011). Analizando el cambio climático a partir de los glaciares del Ecuador [in Spanish]..

[r43] Frazzoli CO, Orisakewe OE, Dragone R, Mantovani A (2010). Diagnostic health risk assessment of electronic waste on the general population in developing countries scenarios.. Environ Impact Assess Rev.

[r44] Garcia S, Mercer R (2003). Experiencia Latinoamericana. Salud infantil y plomo en Argentina [in Spanish].. Salud Publica Mex.

[r45] Gavilán-García A, Román-Moguel G, Almada-Calvo F, Aburto-Mejía S (2009). Electronic waste generation and technology for an appropriate management in Mexico.. Bol Soc Quím Méx.

[r46] Gibb H O’Leary KG 2014 Mercury exposure and health impacts among individuals in the artisanal and small-scale gold mining community: a comprehensive review. Environ Health Perspect 122 667 672; 10.1289/ehp.1307864 24682486 PMC4080518

[r47] González-Andrade F, López-Pulles R, Estévez E (2010). Acute pesticide poisoning in Ecuador: a short epidemiological report.. J Public Health.

[r48] Grandjean P, White RF, Nielsen A, Cleary D, de Oliveira Santos EC (1999). Methylmercury neurotoxicity in Amazonian children downstream from gold mining.. Environ Health Perspect.

[r49] Gunsilius E, Spies S, García-Cortés S, Medina M, Dias S, Scheinberg A, et al. (2011). Recovering Resources, Creating Opportunities: Integrating the Informal Sector into Solid Waste Management.. http://www.giz.de/de/downloads/giz2011-en-recycling-partnerships-informal-sector-final-report.pdf.

[r50] Haggerty R, Rothman J. (1975). Child Health and the Community..

[r51] Handal AJ Lozoff B Breilh J Harlow SD 2007 Effect of community of residence on neurobehavioral development in infants and young children in a flower-growing region of Ecuador. Environ Health Perspect 115 128 133; 10.1289/ehp.9261 17366832 PMC1797846

[r52] Harari R, Harari H (2006). Children’s environment and health in Latin América: the Ecuadorian case.. Ann NY Acad Sci.

[r53] Harari R Julvez J Murata K Barr D Bellinger DC Debes F 2010 Neurobehavioral deficits and increased blood pressure in school-age children prenatally exposed to pesticides. Environ Health Perspect 118 890 896; 10.1289/ehp.0901582 20185383 PMC2898869

[r54] Henao S, Arbelaez MP (2002). Epidemiologic situation of acute pesticide poisoning in Central America, 1992–2000.. Epidemiol Bull.

[r55] Hernández-Morales AL, Zonana-Nacach A, Zaragoza-Sandoval VM (2009). Associated risk factors in acute leukemia in children. A cases and controls study [in Spanish].. Rev Med Inst Mex Seguro Soc.

[r56] Hopenhayn-Rich C, Browning SR, Hertz-Picciotto I, Ferreccio C, Peralta C, Gibb H (2000). Chronic arsenic exposure and risk of infant mortality in two areas of Chile.. Environ Health Perspect.

[r57] Hrubá F, Strömberg U, C˘erná M, Chen C, Harari F, Harari R (2012). Blood cadmium, mercury, and lead in children: an international comparison of cities in six European countries, and China, Ecuador, and Morocco.. Environ Int.

[r58] Huo X Peng L Xu X Zheng L Qiu B Qi Z 2007 Elevated blood lead levels of children in Guiyu, an electronic waste recycling town in China. Environ Health Perspect 115 1113 1117; 10.1289/ehp.9697 17637931 PMC1913570

[r59] IARC (International Agency for Research on Cancer). (2012). Arsenic, metals, fibres, and dusts.. IARC Monogr Eval Carcinog Risk Hum 100C.

[r60] Jenkins R. (1998). Industrialization, Trade and Pollution in Latin America: A Review of the Issues.. http://lasa.international.pitt.edu/LASA98/Jenkins.pdf.

[r61] Juanena C, Battocletti A, Carballal L, Tarán L. (2011). Intoxicaciones en menores de 15 años [in Spanish].

[r62] Kordas K, Queirolo EI, Ettinger AS, Wright RO, Stoltzfus RJ (2010). Prevalence and predictors of exposure to multiple metals in preschool children from Montevideo, Uruguay.. Sci Total Environ.

[r63] Laborde A (2004). New roles for poison control centres in the developing countries.. Toxicology.

[r64] Laborde A, Martínez L, Martínez-López W, Méndez-Acuña L, Morador MJ, Fuster T (2006). Clinical assessment and genotoxicity biomarkers in a population of children and adults exposed to multiple pesticides [in Spanish].. Acta Toxicol Argent.

[r65] Landrigan PJ, Goldman LR (2011). Children’s vulnerability to toxic chemicals: a challenge and opportunity to strengthen health and environmental policy.. Health Aff (Millwood).

[r66] Landrigan PL, Suk WA (2015). Jenny Pronczuk de Garbino: a Global Champion for Children’s Health.. Environ Health Perspect.

[r67] Li Y, Xu X, Liu J, Wu K, Gu C, Shao G (2008a). The hazard of chromium exposure to neonates in Guiyu of China.. Sci Total Environ.

[r68] Li Y, Xu X, Wu K, Chen G, Liu J, Chen S (2008b). Monitoring of lead load and its effect on neonatal behavioral neurological assessment scores in Guiyu, an electronic waste recycling town in China.. J Environ Monit.

[r69] Lim SS, Vos T, Flaxman AD, Danaei G, Shibuya K, Adair-Rohani H (2012). A comparative risk assessment of burden of disease and injury attributable to 67 risk factor clusters in 21 regions, 1990–2010: a systematic analysis for the Global Burden of Disease Study 2010.. Lancet.

[r70] Liu J, Xu X, Wu K, Piao Z, Huang J, Guo Y (2011). Association between lead exposure from electronic waste recycling and child temperament alterations.. Neurotoxicology.

[r71] Lundgren K. (2012). The Global Impact of E-waste: Addressing the Challenge. Geneva:Programme on Safety and Health at Work and the Environment (SafeWork), Sectoral Activities Department (SECTOR) International Labour Office.. http://www.ilo.org/wcmsp5/groups/public/---ed_dialogue/---sector/documents/publication/wcms_196105.pdf.

[r72] Mañay N, Cousillas AZ, Alvarez C, Heller T (2008). Lead contamination in Uruguay: the “La Teja” neighborhood case.. Rev Environ Contam Toxicol.

[r73] Manzanares-Acuña E, Vega-Carrilo HR, Salas-Luévano MA, Hernándes-Dávilla VM, Letechipía-de Léon C, Bañuelos-Valenzuela R (2006). Niveles de plomo en la población de alto riesgo y su entorno en San Ignacio, Fresnillo, Zacatecas, México [in Spanish].. Salud Publica Mex.

[r74] Marinho FM, Soliz P, Gawryszewski V, Gerger A (2013). Epidemiological transition in the Americas: changes and inequalities [Abstract].. Lancet.

[r75] McDiarmid MA, Gehle K (2006). Preconception brief: occupational and environmental exposures.. Matern Child Health J.

[r76] McMichael AJ, Campbell-Lendrum D, Kovats S, Edwards S, Wilkinson P, Wilson T, et al. (2004). Global climate change. In: Comparative Quantification of Health Risks. Global and Regional Burden of Disease Attributable to Selected Major Risk Factors. Geneva:WHO.. http://www.who.int/publications/cra/chapters/volume2/1543-1650.pdf?ua=1.

[r77] Ministerio de Salud, Comision Nacional del Medio Ambiente, Gobierno de Chile. (2009). Guia técnica sobre manejo de baterías de plomo acido usadas [in Spanish].. http://www.sinia.cl/1292/articles-47018_recurso_1.pdf.

[r78] Ministerio de Salud Publica, Republica Oriental del Uruguay. (2011). Pautas de manejo y seguimiento de población pediátrica según valores de plomo en sangre [in Spanish].. http://www.msp.gub.uy/sites/default/files/Anexo_Resolucion_123.pdf.

[r79] Ministerio de Salud, Republica de Argentina. (2009). Salud Publica Resolución 436/2009. Apruébese el Instructivo para el Cumplimiento de la Resolución del Ministerio de Salud de la Nación Nº 7/2009 que estableció un límite de plomo permitido en pinturas, lacas y barnices [in Spanish].. http://www.inti.gob.ar/certificaciones/pdf/res436_2009.pdf.

[r80] Ministerio de Salud, Republica de Argentina. (2013). Guía De Prevención, Diagnóstico, Tratamiento Y Vigilancia Epidemiológica De Las Intoxicaciones Ambientales Infantiles con Plomo [in Spanish].. http://www.msal.gov.ar/images/stories/bes/graficos/0000000293cnt-guia_intoxicaciones_con_plomo_2013.pdf.

[r81] Ministerio de Salud Publica, Republica del Peru. (2007). Guía técnica: guía de práctica clínica para el manejo de pacientes con intoxicación por plomo [in Spanish].. http://www.sis.gob.pe/ipresspublicas/normas/pdf/minsa/GUIASPRACTICAS/2007/RM511_2007.pdf.

[r82] Ministerio del Ambiente, República del Ecuador. (2003). Cambio Climático [in Spanish].. http://unfccc.int/resource/docs/natc/ecunc1s.pdf.

[r83] Ministerio del Ambiente, República del Ecuador. (2012). Estrategia Nacional de Cambio Climático del Ecuador: ENCC 2012–2025 [in Spanish].. http://www.redisas.org/pdfs/ENCC.pdf.

[r84] Monge P, Wesseling C, Guardado J, Lundberg I, Ahlbom A, Cantor KP (2007). Parental occupational exposure to pesticides and the risk of childhood leukemia in Costa Rica.. Scand J Work Environ Health.

[r85] Mutanen P, Hemminki K (2001). Childhood cancer and parental occupation in the Swedish Family-Cancer Database.. J Occup Environ Med.

[r86] MVOTMA (Ministerio de Vivienda, Ordinamiente Teritorial y Medio Ambiente, Republica Oriental del Uruguay). (2011). Decreto 69/011—Contenido de plomo en pinturas [in Spanish].. http://www.mvotma.gub.uy/ciudadania/tramites/tramites-medio-ambiente/item/10004953-contenido-de-plomo-en-pinturas.html.

[r87] MVOTMA (Ministerio de Vivienda, Ordinamiente Teritorial y Medio Ambiente, Republica Oriental del Uruguay). (2012). Decreto 373/003—Reglamento de baterías plomo acido usadas o a ser desechadas [in Spanish].. http://mvotma.gub.uy/planes-de-mejora-del-desempeno-ambiental/itemlist/tag/Baterías.html.

[r88] Naujokas MF Anderson B Ahsan H Aposhian HV Graziano JH Thompson C 2013 The broad scope of health effects from chronic arsenic exposure: update on a worldwide public health problem. Environ Health Perspect 121 295 302; 10.1289/ehp.1205875 23458756 PMC3621177

[r89] Noel-Brune M Goldizen FC Neira M van den Berg M Lewis N King M 2013 Health effects of exposure to e-waste [Letter]. Lancet Glob Health 1 e70; 10.1016/S2214-109X(13)70020-2 25104155

[r90] Noyes PD, McElwee MK, Miller HD, Clark BW, Van Tiem MA, Walcott KC (2009). The toxicology of climate change: environmental contaminants in a warming world.. Environ Int.

[r91] NRC (National Research Council). (1993). Pesticides in the Diets of Infants and Children. Washington, DC:National Academies Press.. http://www.nap.edu/openbook.php?isbn=0309048753.

[r92] PAHO (Pan American Health Organization). (2007). Core Health Data System—Glossary.. http://www1.paho.org/English/SHA/glossary.htm.

[r93] PAHO (Pan American Health Organization). (2011). The Atlas of Children’s Health and Environment in the Americas.

[r94] PAHO (Pan American Health Organization). (2012a). Agua y saneamiento. Evidencias para políticas públicas con enfoque en derechos humanos y resultados en salud pública [in Spanish].. http://www.paho.org/tierra/images/pdf/agua_y_saneamiento_web.pdf.

[r95] PAHO (Pan American Health Organization). (2012b). The environment and human security. In: Health in the Americas.. http://www.paho.org/saludenlasamericas/index.php?option=com_content&view=article&id=56&Itemid=52&lang=en.

[r96] PAHO (Pan American Health Organization), WHO (World Health Organization). (2007). La equidad en la mira: La salud pública en Ecuador durante las últimas décadas [in Spanish].. http://www.paho.org/ecu/index.php?option=com_docman&task=doc_download&gid=58&Itemid=.

[r97] Patz JA, Epstein PR, Burke TA, Balbus JM (1996). Global climate change and emerging infectious diseases.. JAMA.

[r98] Paul M, Himmelstein J (1988). Reproductive hazards in the workplace: what the practitioner needs to know about chemical exposures.. Obstet Gynecol.

[r99] Pawlas N, Strömberg U, Carlberg B, Cerna M, Harari F, Harari R (2013). Cadmium, mercury and lead in the blood of urban women in Croatia, the Czech Republic, Poland, Slovakia, Slovenia, Sweden, China, Ecuador and Morocco.. Int J Occup Med Environ Health.

[r100] Pinault LL Hunter FF 2011 New highland distribution records of multiple *Anopheles* species in the Ecuadoran Andes. Malar J 10 236; 10.1186/1475-2875-10-236 21835004 PMC3176254

[r101] Pronczuk J, Bruné MN, Gore F. (2011). Children’s environmental health in developing countries. In: Encyclopedia of Environmental Health (Nriagu JO, ed).

[r102] Prüss-Üstün A, Corvalán C. (2006). Preventing Disease through Healthy Environments: Towards an Estimate of the Environmental Burden of Disease. Geneva:WHO.. http://www.who.int/quantifying_ehimpacts/publications/preventingdisease.pdf.

[r103] Recio-Vega R, Valdez-Abrego C, Adame-Lopez B, Gurrola-Mendez A (2012). Surveillance of elevated blood lead levels in children in Torreon, Coahuila, Mexico, 1998–2010.. Int J Hyg Environ Health.

[r104] Rodríguez S, Mallet J, Laborde A (2007). Intoxicación aguda por diazinón en niños [in Spanish].. Arch Pediatr Urug.

[r105] Rojas M, Espinosa C, Seijas D (2003). Association between blood lead and sociodemographic parameters among children [in Spanish].. Rev Saude Publica.

[r106] Romieu I, Barraza-Villarreal A, Escamilla-Nuñez C, Almstrand AC, Diaz-Sanchez D, Sly PD, et al. (2008). Exhaled breath malondialdehyde as a marker of effect of exposure to air pollution in children with asthma.. J Allergy Clin Immunol.

[r107] Romieu I, Carreon T, Lopez L, Palazuelos E, Rios C, Manuel I (1995). Environmental urban lead exposure and blood lead levels in children of Mexico City.. Environ Health Perspect.

[r108] Romieu I, Ramirez Aguilar M, Moreno Marcias H, Barraza Villarreal A, Hernandez Cadena L, Arroyo LC. (2003). Health Impacts of Air Pollution on Morbidity and Mortality Among Children of Ciudad Juárez, Chihuahua, Mexico.. http://www3.cec.org/islandora/en/item/2024-health-impacts-air-pollution-morbidity-and-mortality-among-children-ciudad-en.pdf.

[r109] Rosado JL Ronquillo D Kordas K Rojas O Alatorre J Lopez P 2007 Arsenic exposure and cognitive performance in Mexican schoolchildren. Environ Health Perspect 115 1371 1375; 10.1289/ehp.9961 17805430 PMC1964916

[r110] Rubio-Andrade M Valdés-Pérezgasga F Alonso J Rosado JL Cabrián ME Garcia-Vargas GG 2011 Follow-up study on lead exposure in children living in a smelter community in northern Mexico. Environ Health 10 66; 10.1186/1476-069X-10-66 21767395 PMC3152872

[r111] Sánchez-Cortez J, Ilabaca-Marileo M, Martín MA, de los Angeles Viñas M, Bravo-Méndez R (2003). Prevalencia de plomo en sangre, en niños escolares de Santiago de Chile [in Spanish].. Salud Publica Mex.

[r112] Schütz A, Barregård L, Sällsten G, Wilske J, Manay N, Pereira L (1997). Blood lead in Uruguayan children and possible sources of exposure.. Environ Res.

[r113] Sciedeck D, Sundelin B, Readman JW, Macdonald RW (2007). Interactions between climate change and contaminants.. Mar Pollut Bull.

[r114] Seijas D, Squillante G (2008). Plomo em sangre, estado nutricional y estratificacion socioeconômica, em niños de uma comunidad de Valencia [in Spanish].. Anales Venezoelanos de Nutrición.

[r115] Sheffield PE Landrigan PJ 2011 Global climate change and children’s health: threats and strategies for prevention. Environ Health Perspect 119 291 298; 10.1289/ehp.1002233 20947468 PMC3059989

[r116] Sly PD, Neira M, Collman G, Carpenter DO, Landrigan PJ, Van Den Berg M (2014). Networking to advance progress in children’s environmental health.. Lancet Glob Health.

[r117] Smith AH Marshall G Liaw J Yuan Y Ferreccio C Steinmaus C 2012 Mortality in young adults following *in utero* and childhood exposure to arsenic in drinking water. Environ Health Perspect 120 1527 1531; 10.1289/ehp.1104867 22949133 PMC3556614

[r118] Smith KR, McCracken JP, Weber MW, Hubbard A, Jenny A, Thompson LM (2011). Effect of reduction in household air pollution on childhood pneumonia in Guatemala (RESPIRE): a randomised controlled trial.. Lancet.

[r119] Soares da Silva A, Meyer MA, Mujica OJ. (2013). Exploratory Analysis of Social Gradients and Inequalities in the Use of Solid Fuels in Latin America and the Caribbean [Poster]. Proceedings of the American Public Health Association (APHA). 287890. 141st Annual Meeting and Expo, 2–6 November 2013. Boston, MA.. https://apha.confex.com/apha/141am/webprogram/Paper287890.html.

[r120] Souza MS, Magnarelli GG, Rovedatti MG, Santa Cruz S, Pechen De D’Angelo AM (2005). Prenatal exposure to pesticides: analysis of human placental acetylcholinesterase, glutathione *S*-transferase and catalase as biomarkers of effect.. Biomarkers.

[r121] Souza MS, Rovedatti MG, Sánchez S, Santa Cruz S, Pechén de D’Angelo AM, Magnarelli G (2006). Biomarkers for monitoring in utero exposure to pesticides in a rural community [in Spanish].. Acta Toxicol Argent.

[r122] Steinmaus CM, Ferreccio C, Romo JA, Yuan Y, Cortes S, Marshall G (2013). Drinking water arsenic in northern Chile: high cancer risks 40 years after exposure cessation.. Cancer Epidemiol Biomarkers Prev.

[r123] Steubing B, Böni H, Schluep M, Silva U, Ludwig C (2010). Assessing computer waste generation in Chile using material flow analysis.. Waste Manag.

[r124] Suk WA Ruchirawat KM Balakrishnan K Berger M Carpenter D Damstra T 2003 Environmental threats to children’s health in Southeast Asia and the Western Pacific. Environ Health Perspect 111 1340 1347; 10.1289/ehp.6059 12896856 PMC1241616

[r125] Tomasina F, Laborde A. (2010). Contaminantes orgánicos persistentes y plaguicidas [in Spanish]. REDESMA 4.. http://www.dso.fmed.edu.uy/sites/www.dso1.fmed.edu.uy/files/materiales/REDESMA_09_art03.pdf.

[r126] UN (United Nations). (2011). 2011 High-Level Meeting on Prevention and Control of Non-communicable Diseases. General Assembly, United Nations, New York, 19–20 September 2011.. http://www.un.org/en/ga/ncdmeeting2011/.

[r127] UN (United Nations). (2012). StEP Annual Report 2011.. http://www.step-initiative.org/index.php/newsdetails/items/step-annual-report-2011-now-available-157.html.

[r128] UN (United Nations). (2013). Pollutant Release and Transfer Registers.. http://www.unitar.org/cwm/prtr.

[r129] UN Habitat (United Nations Program for Human Settlement). (2012). Estado de las Ciudades de América Latina y el Caribe 2012: Rumbo a una nueva transición urbana. Nairobi, Kenya:UN Habitat.. http://www.onuhabitat.org/index.php?option=com_docman&task=doc_download&gid=816&Itemid=18.

[r130] UNEP (United Nations Environment Programme). (2010). Analysis of Trade Flows and Review of Environmentally Sound Management Practices Related to Products Containing Cadmium, Lead, and Mercury in Latin America and the Caribbean.. http://www.unep.org/chemicalsandwaste/Portals/9/Lead_Cadmium/docs/Trade_Reports/LAC/Trade_report_LAC_English.pdf.

[r131] van Wendel de Joode B, Barraza D, Ruepert C, Mora AM, Córdoba L, Oberg M (2012). Indigenous children living nearby plantations with chlorpyrifos-treated bags have elevated 3,5,6-trichloro-2-pyridinol (TCPy) urinary concentrations.. Environ Res.

[r132] Vega-Dienstmaier JM, Salinas-Piélago JE, del Rosario Gutiérrez-Campos M, Mandamiento-Ayquipa RD, del Carmen Yara-Hokama M, Ponce-Canchihuamán J (2006). Lead levels and cognitive abilities in Peruvian children.. Rev Bras Psiquiatr.

[r133] Virta RL. (2009). World Asbestos Consumption from 2003 through 2007. Reston, VA:U.S. Geological Survey.. http://minerals.usgs.gov/minerals/pubs/commodity/asbestos/mis-2007-asbes.pdf.

[r134] Virta RL. (2012). Asbestos [Advance Release]. In: 2012 Minerals Yearbook.. http://minerals.usgs.gov/minerals/pubs/commodity/asbestos/myb1-2012-asbes.pdf.

[r135] von Ehrenstein OS, Poddar S, Yuan Y, Mazumder DG, Eskenazi B, Basu A (2007). Children’s intellectual function in relation to arsenic exposure.. Epidemiology.

[r136] Wade L (2013). Mercury pollution. Gold’s dark side.. Science.

[r137] Wasserman GA Liu X Parvez F Ahsan H Factor-Litvak P van Geen A 2004 Water arsenic exposure and children’s intellectual function in Araihazar, Bangladesh. Environ Health Perspect 12 1329 1333; 10.1289/ehp.6964 15345348 PMC1247525

[r138] Wesseling C, Corriols M, Bravo V (2005). Acute pesticide poisoning and pesticide registration in Central America.. Toxicol Appl Pharmacol.

[r139] Wesseling C, McConnell R, Partanen T, Hogstedt C (1997). Agricultural pesticide use in developing countries: health effects and research needs.. Int J Health Serv.

[r140] WHO (World Health Organization). (2000). Global Water Supply and Sanitation Assessment 2000 Report. Geneva:WHO.. http://www.who.int/water_sanitation_health/monitoring/globalassess/en/.

[r141] WHO (World Health Organization). (2010a). Childhood Lead Poisoning.. http://www.who.int/ceh/publications/leadguidance.pdf.

[r142] WHO (World Health Organization). (2010b). Children’s Environmental Health Units.. http://www.who.int/ceh/publications/childrensunit.pdf.

[r143] WHO (World Health Organization). (2010c). Global Plan of Action for Children’s Health and the Environment (2010–2015).. http://www.who.int/ceh/cehplanaction10_15.pdf?ua=1.

[r144] WHO (World Health Organization). (2010d). International Programme on Chemical Safety. Ten Chemicals of Major Public Health Concern.. http://www.who.int/ipcs/assessment/public_health/chemicals_phc/en/.

[r145] WHO (World Health Organization). (2012). The Paediatric Environmental History. Recording Children’s Exposure to Environmental Health Threats: A “Green Page” in the Medical Record.. http://www.who.int/ceh/capacity/paedenvhistory/en/index.html.

[r146] WHO (World Health Organization). (2014a). 7 Million Premature Deaths Annually Linked to Air Pollution.. http://www.who.int/mediacentre/news/releases/2014/air-pollution/en/.

[r147] WHO (World Health Organization). (2014b). Burden of Disease from Household Air Pollution for 2012. Summary of Results.. http://www.who.int/phe/health_topics/outdoorair/databases/HAP_BoD_results_March2014.pdf?ua=1.

[r148] WHO (World Health Organization). (2014c). Global Health Observatory Repository: Data by Country.. http://apps.who.int/gho/data/node.country.

[r149] WHO (World Health Organization). (2014d). Public Health, Environmental and Social Determinants of Health (PHE). Ambient (Outdoor) Air Pollution in Cities Database 2014.. http://www.who.int/phe/health_topics/outdoorair/databases/cities/en/.

[r150] WHO (World Health Organization), UNEP (United Nations Environment Programme). (2010). Healthy Environments for Healthy Children: Key Messages for Action. 2010. http://www.who.int/entity/ceh/publications/hehc_booklet_en.pdf?ua=1.

[r151] Wichmann FA, Müller A, Busi LE, Cianni N, Massolo L, Schlink U (2009). Increased asthma and respiratory symptoms in children exposed to petrochemical pollution.. J Allergy Clin Immunol.

[r152] World Bank. (2012). Living Standards Measurement Survey. Guatemala 2000. ENCOVI Survey.. http://go.worldbank.org/D2VAFINBG0.

[r153] Wu K, Xu X, Liu J, Guo Y, Li Y, Huo X (2010). Polybrominated diphenyl ethers in umbilical cord blood and relevant factors in neonates from Guiyu, China.. Environ Sci Technol.

[r154] Xu X, Yang H, Chen A, Zhou Y, Wu K, Liu J (2012). Birth outcomes related to informal e-waste recycling in Guiyu, China.. Reprod Toxicol.

[r155] Yang H, Huo X, Yekeen TA, Zheng Q, Zheng M, Xu X (2013). Effects of lead and cadmium exposure from electronic waste on child physical growth.. Environ Sci Pollut Res Int.

[r156] Zheng G, Xu X, Li B, Wu K, Yekeen TA, Huo X (2013). Association between lung function in school children and exposure to three transition metals from an e-waste recycling area.. J Expo Sci Environ Epidemiol.

[r157] Zoeteman BC, Krikke HR, Venselaar J (2010). Handling WEEE waste flows: on the effectiveness of producer responsibility in a globilizing world.. Int J Adv Manuf Technol.

