# Supplementary material for: Children’s Health in Latin America: The Influence of Environmental Exposures
Source: Environ Health Perspect. 2014 Dec 5;123(3):201–9. doi: 10.1289/ehp.1408292 (PMC4348745; doi:10.1289/ehp.1408292)
Supplement: (207 KB) PDF [file ehp.1408292.s001.508.pdf]

Environmental Health Perspectives doi:10.1289/ehp.1408292

Supplemental Material

Children's Health in Latin America: The Influence of Environmental Exposures

Amalia Laborde, Fernando Tomasina, Fabrizio Bianchi, Marie-Noel Bruné, Irena Buka, Pietro Comba, Lilian Corra, Liliana Cori, Christin Maria Duffert, Raul Harari, Ivano Iavarone, Melissa A. McDiarmid, Kimberly A. Gray, Peter D. Sly, Agnes Soares, William A. Suk, and Philip J. Landrigan

Appendix S1. International and regional meetings on children's environmental health convened by WHO.

- **2002. First International Conference on Environmental Threats to the Health of Children: Hazards and Vulnerability, Bangkok, Thailand.** This conference was held in partnership with the Chulabhorn Research Institute. A major outcome was the Bangkok Statement on Children's Environmental Health, which established priorities for action and a commitment to national and international activities in the area of children's environmental health (Suk et al. 2003).
- **2005. Mar del Plata Conference on Children's Health and the Environment, Mar del Plata, Argentina.** Convened by WHO and PAHO in collaboration with the Argentine Pediatric Society, this conference produced the declaration of Mar del Plata in which Ministries of Health and national pediatric societies in seven countries of the Southern Cone of South America pledged to make the protection of children against environmental threats to health a high national priority (HEMA 2005).
- **2005. Second International Conference on Children's Environmental Health: Healthy Environments, Healthy Children: Increasing Knowledge and Taking Action, Buenos Aires, Argentina.** WHO and PAHO convened this conference. A consequence was creation of a network of Centers of Excellence in Children's Environmental Health (Unidades de Pediatria Ambiental) across Latin America. These Centers, known in North America as Pediatric Environmental Health Specialty Units, now exist in Argentina, Uruguay, Chile, and Mexico as well as in the United States, Canada, and Spain (WHO 2010b, Wilborne-Davis et al. 2007).

- **2009. Third International Conference on Children's Health and the Environment: From Research and Knowledge to Policy and Action, Busan, Republic of Korea.** More than 600 participants from 60 countries met in Busan in June 2009. The Busan Pledge for Action on Children's Health and the Environment, which emerged from the conference, called for a global plan of action to improve children's environmental health in countries around the world (Gavidia et al. 2010).
- **2012. International Scientific Conference: Environmental Health in the Political Agenda. Montevideo, Uruguay on March 22-24, 2012.** This conference was convened to continue implementation of the WHO/PAHO strategy on children's environmental health and to build regional capacity in Latin America. (http://www.dso.fmed.edu.uy/sites/www.dso1.fmed.edu.uy/files/materiales/1_pdfsam_LIBRO_RESUMENES-_Conferencia_Salud_Ambiental-1.pdf (accessed 6 October 2014).

Appendix S2. Accomplishments of the WHO/PAHO program in children's environmental health

- Development of a training tool, the WHO Training Package on children's environmental health for the Health Sector (WHO 2011), followed by implementation of training workshops in countries around the world based on the Training Package.
- Construction of the Green Page for environmental health history taking (WHO 2012).
- Incorporation of a children's environmental health component, based on the Green Page, into WHO's Integrated Management of Childhood Illnesses (WHO 2010c; Pronczuk et al. 2011; WHO 2012).
- Promotion and international coordination of longitudinal epidemiological studies of children's health and the environment (Golding et al. 2009).
- Development of environmental health indicators for use in countries around the world (WHO 2009).
- Promotion of cooperative research, e.g., in childhood asthma; arsenic exposure in pregnant women and their children in Thailand; and asthma and air pollution in urban areas in India (WHO/UNEP 2010; Pronczuk et al. 2011).
- Creation of national profiles of children's health and the environment utilizing the WHO Multiple Exposures Multiple Effects Model (Corra 2008).
- Contributions to Global Burden of Disease project, showing, for example, that lead is responsible for approximately 1% of the global burden of disease (Prüss-Ustün et al. 2011; Fewtrell et al. 2004).
- Construction of strong proactive partnerships with non-governmental organizations such as the International Society of Doctors for the Environment (ISDE), the International

Network on Children's Health Environment and Safety (INCHES) and the Nurses Network (WHO/UNEP 2010; WHO 2010c; Pronczuk et al. 2011).

- Collaboration with UN Environmental Programme to remove lead from gasoline worldwide and to develop a children's environmental health newsletter (WHO and UNEP 2013).
- Produced many publications in multiple languages on topics in children's environmental health (WHO 2010a, 2013a, 2013b).
- Inception of a global network of Collaborating Centres in Children's Environmental Health (Sly et al. 2014).

References

Corra L. 2008. Children’s Environmental Health National Profile: A multisectorial stakeholder collaboration experience in Argentina. Int J Environment and Health 2:304–313.

Gavidia T, Brune MN, McCarty KM, Pronczuk J, Etzel R, Neira M, et al. 2010. Children’s environmental health – from knowledge to action. The Lancet. 377:1134-1136.

HEMA (Health and Environment Ministers of the Americas). 2005. Declaration of Mar del Plata. Available at: http://www.oas.org/hema/english/Documents/MarDelPlataEng.doc [accessed 8 November 2013].

Fewtrell LJ, Prüss-Ustün A, Landrigan P, Ayuso-Mateos JL. 2004. Estimating the global burden of disease of mild mental retardation and cardiovascular diseases from environmental lead exposure. Environ Res 94:120-133.

Golding J, Birmingham K, Jones R. 2009. Special issue: a guide to undertaking a birth cohort study: purposes, pitfalls and practicalities. Paediatric and Perinatal Epidemiology 23(Suppl s1):1-236.

Pronczuk J, Bruné M-N, Gore F. 2011. Children's Environmental Health in Developing Countries. In: Encyclopedia of Environmental Health (Nriagu JO, ed) Burlington:Elsevier 601-610. http://dx.doi.org/10.1016/B978-0-444-52272-6.00008-8 (http://www.sciencedirect.com/science/article/pii/B9780444522726000088)

Prüss-Ustün A, Vickers C, Haefliger P, Bertollini R. 2011. Knowns and unknowns on burden of disease due to chemicals: a systematic review. Environ Health 10:1-15.

Sly PD, Neira M, Collman G, Carpenter DO, Landrigan PJ, Van Den Berg M et al. 2014. Networking to advance progress in children's environmental health. The Lancet Global Health 2 (3):e129-e130.

Suk WA, Ruchirawat KM, Balakrishnan K, Berger M, Carpenter D, Damstra T, et al. 2003. Environmental threats to children’s health in Southeast Asia and the western Pacific. Environ Health Perspect 111:1340-1347.

WHO (World Health Organization). 2009. Children’s Environmental Health Indicators: Presenting Regional Successes – Learning for the Future. Geneva:World Health Organization.

WHO (World Health Organization). 2010a. Asbestos: Elimination of Asbestos-Related Diseases. Fact Sheet No 343. Available: http://www.who.int/mediacentre/factsheets/fs343/en/index.html [accessed 12 November 2013].

WHO (World Health Organization). 2010b. Children's Environmental Health Units. Available: http://www.who.int/ceh/publications/childrensunit.pdf [Accessed 8 November 2013].

WHO (World Health Organization). 2010c. Global Plan of Action for Children's Health and the Environment (2010-2015). Available at: http://www.who.int/ceh/cehplanaction10_15.pdf?ua=1 [accessed 24 April 2014]

WHO (World Health Organization). 2011. Training Package on CEH for the Health Sector. Available: http://www.who.int/ceh/capacity/trainpackage/en/index.html [accessed 8 November 2013].

WHO (World Health Organization). 2012. Green Page. Available: http://www.who.int/ceh/capacity/paedenvhistory/en/index.html [accessed 8 November 2013].

WHO (World Health Organization). 2013a. Millennium Development Goals: Progress towards the Health-Related Millennium Development Goals. Fact Sheet No 290. Available: http://www.who.int/mediacentre/factsheets/fs290/en/ [accessed 12 November 2013.]

WHO (World Health Organization). 2013b. Obesity and Overweight. Fact Sheet No 311. Available: http://www.who.int/mediacentre/factsheets/fs311/en/ [accessed 12 November 2013.]

WHO (World Health organization), UNEP (United Nations Environment Programme). Healthy environments for healthy children: key messages for action. 2010 (http://www.who.int/ceh/publications/hehc_booklet/en/) [accessed 24 April 2014]

WHO (World Health Organization), UNEP (United Nations Environment Programme). 2013. Healthy Environments for Children Alliance Newsletter. Available: http://www.who.int/heca/infomaterials/hecanet/en/ [accessed 12 November 2013].

Wilborne-Davis P, Kirkland KH, Mulloy KB. 2007. A model for physician education and consultation in pediatric environmental health: the Pediatric Environmental Health Specialty Units (PEHSU) program. Pediatr Clin North Am 54:1-13.
